# Transcriptome and Metabolome Profiling Reveal the Resistance Mechanisms of Rice against Brown Planthopper

**DOI:** 10.3390/ijms23084083

**Published:** 2022-04-07

**Authors:** Qian Zhang, Tianzhu Li, Mingyang Gao, Meng Ye, Manxia Lin, Di Wu, Jianping Guo, Wei Guan, Jing Wang, Ke Yang, Lili Zhu, Yichen Cheng, Bo Du, Guangcun He

**Affiliations:** State Key Laboratory of Hybrid Rice, College of Life Sciences, Wuhan University, Wuhan 430072, China; qinazhang@whu.edu.cn (Q.Z.); 2020102040042@whu.edu.cn (T.L.); gmy981109@whu.edu.cn (M.G.); 2021202040085@whu.edu.cn (M.Y.); 2021282040185@whu.edu.cn (M.L.); 2014202040076@whu.edu.cn (D.W.); guojianping0521@163.com (J.G.); 2015202040081@whu.edu.cn (W.G.); whuwangjingxz@163.com (J.W.); 2019102040039@whu.edu.cn (K.Y.); zhulili58@sina.com (L.Z.); 13872209530@163.com (Y.C.)

**Keywords:** rice, BPH, transcriptome, metabolome, IAA, epigallocatechin

## Abstract

Brown planthopper (*Nilaparvata lugens Stål*, BPH) is one of the most destructive insects affecting rice production. To better understand the physiological mechanisms of how rice responds to BPH feeding, we analyzed BPH-induced transcriptomic and metabolic changes in leaf sheaths of both BPH-susceptible and -resistant rice varieties. Our results demonstrated that the resistant rice reduced the settling, feeding and growth of BPH. Metabolic analyses indicated that BPH infestation caused more drastic overall metabolic changes in the susceptible variety than the resistant rice. Differently accumulated metabolites (DAMs) belonging to flavonoids were downregulated in the susceptible rice but upregulated in resistant variety. Transcriptomic analyses revealed more differentially expressed genes (DEGs) in susceptible rice than resistant rice, and DEGs related to stimulus were significantly upregulated in resistant rice but downregulated in susceptible rice. Combined analyses of transcriptome and metabolome showed that many DEGs and DAMs were enriched in phenylpropane biosynthesis, flavonoid biosynthesis, and plant hormone signal transduction. We conducted correlation analyses of DEGs and DAMs in these pathways and found a high correlation between DEGs and DAMs. Then, we found that the contents of endogenous indole 3-acetic acid (IAA) in resistant rice was lower than that of susceptible rice after BPH feeding, while the salicylic acid (SA) content was the opposite. For functional analysis, an exogenous application of IAA decreased rice resistance to BPH, but the exogenous application of SA increased resistance. In addition, biochemical assessment and quantitative PCR analysis showed that the lignin content of resistant accession was constitutively higher than in susceptible accession. By adding epigallocatechin, the substrate of anthocyanidin reductase (ANR), to the artificial diet decreased the performance of BPH. We first combined a transcriptome-metabolome-wide association study (TMWAS) on rice resistance to BPH in this study. We demonstrated that rice promoted resistance to BPH by inducing epigallocatechin and decreasing IAA. These findings provided useful transcriptomic and metabolic information for understanding the rice-BPH interactions.

## 1. Introduction

Rice is an important food crop and suffers various hazards during the growth process [1,2]. BPH is the most notorious insect pest of rice [3,4,5,6,7,8]. BPH infestation can lead to a lower rate of photosynthesis and reduction in organics and, eventually, to the death of susceptible rice [9]. Contrary to this, resistant rice plants carrying the BPH-resistance gene showed minor damage and grew normally under BPH infestation [10]. Therefore, integrating the BPH resistance gene into the cultivated rice varieties is the most economical, effective and sustainable strategy for preventing BPH. Understanding the rice mechanisms that are resistant to BPH is a significant part of developing insect-resistant rice varieties.

Previous studies found that BPH preferred feeding on susceptible rice plants rather than resistant rice; moreover, the weight gain and honeydew secretion of BPH after feeding on susceptible rice were significantly higher than those feeding on insect-resistant rice [11]. Many pieces of evidence revealed gene expression changes in resistant and susceptible rice under BPH attack by cDNA microarray experiment and transcriptome. A cDNA microarray experiment found that there more genes were affected during the infestation of BPH in susceptible rice MH63 than in resistant rice B5 [12]. Furthermore, BPH infestation can cause leaf senescence, mainly due to the decline in photosynthesis in susceptible rice, but only a weak reduction in protein and sucrose contents in resistant rice [9]. Another study identified that the change in the SA content occurred more rapidly in the resistant rice, Rathu Heenati, than in the susceptible rice, Taichung Native 1, and the expression of plant-hormone-related genes were different in these two rice varieties [3,13].

Changes in metabolites in rice can also be observed after BPH infestation by metabolome. Liu et al. compared the metabolic changes in the susceptible rice TN1 and the resistant rice B5 using NMR. The result showed that BPH could lead to significant changes in almost all the core metabolics in TN1, but only GABA shunt and secondary metabolites were affected in B5 [10]. GC-MS conducted by Peng et al. revealed that glyoxylate cycle, fatty acid oxidation, gluconeogenesis and GABA shut were induced by BPH in the TN1 plant; however, only the shikimate pathway was induced in resistant rice YHY15 [14]. Moreover, the sterol biosynthetic pathway in susceptible rice Nipponbare was increased after the attack by BPH, while an increase in the phytol metabolism and wax biosynthesis was observed in R6 plants [15]. Biochemical studies and functional assays confirmed that some metabolites played essential roles in rice resistance to BPH. Lu et al. found that BPH infestation induced the accumulation of serotonin, and the suppression of serotonin caused an increased SA level, leading to rice resistance to BPH, and showing a negative role of serotonin in rice resistance to BPH [16]. In addition, the accumulation of callose in resistant rice prevented BPH infestation; by contrast, BPH infestation activated the callose-hydrolyzing enzymes, leading to the decomposition of callose in susceptible rice [17]. Additionally, 3-nitraphthalic acid and tricin extracted from resistant rice inhibited BPH attack, as well as phenol-amides [18,19,20]. Recently, schaftoside in the artificial diet reduced BPH fecundity [21].

Previous studies mainly focused on a single aspect of rice resistance to BPH [4,6,7]. Since the last century, many studies have focused on discovering resistance genes in rice, and most of these genes belong to NBS-LRR genes [4,7,8,11]. With the development of the transcriptome, more regulatory genes can be found [9,12,13]. Recently, the development of metabolomics enabled the detection of multiple metabolites in plants, bringing great convenience to scientific research [6,10,14,20]. At present, a newly emerging technique, combining transcriptome with widely targeted metabolome analyses, has become a mature technology for providing a comprehensive understanding of how organisms respond to abiotic and biotic stresses [22,23,24,25,26,27]. For example, Kou et al. revealed that the putrescine pathway was involved in potato cold-acclimated freezing tolerance by transcriptome and metabolome analyses [27]. Another study that combined transcriptome and metabolome analyses proved that phytohormones and volatiles played an important role in maize resistance to Asian corn borer [25]. However, there were few studies on the mechanism of rice resistance to BPH.

In this work, we systematically analyzed the metabolome and transcriptome analyses of BPH-infestation-induced changes in a BPH-susceptible and a BPH-resistant rice variety. Our objectives were to find the effective mechanisms in resistant varieties regarding the metabolic and transcriptomic interactions between rice plants and BPH. We also analyzed the BPH-induced changes in the expression of relevant genes and measured the significant metabolites. Finally, we validated the functions of candidate target genes and associated metabolites in rice against BPH. Our work provided a comprehensive overview of rice plant hormones and phenylpropanoid metabolism responses to BPH feeding.

## 2. Results

### 2.1. The Performance of BPH on the Rice Varieties

**Figure 1:**
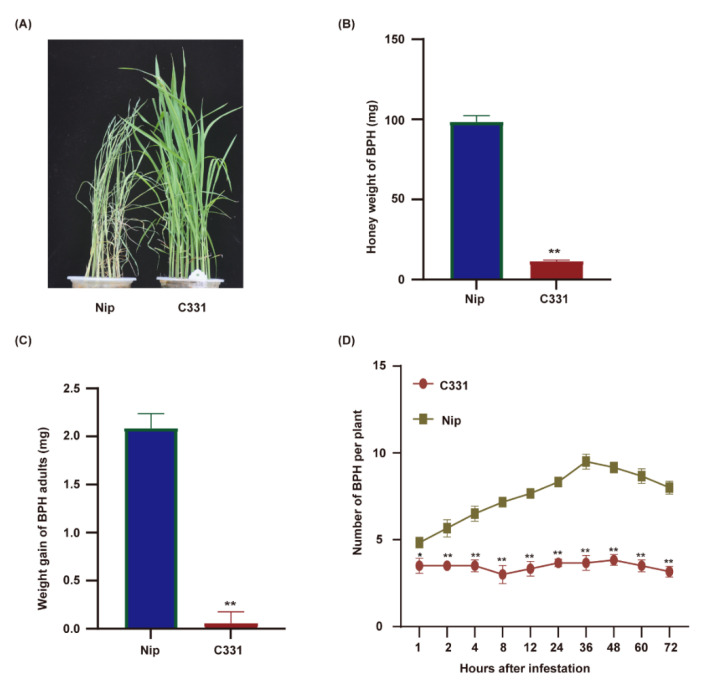
Performance of BPHs on the rice variety Nipponbare and C331. (**A**) BPH-resistance assay of the Nipponbare and C331 rice plants at the seedling stage. 10 BPHs per seeding. (**B**) Honeydew excretion by newly emerged female adult BPHs fed on the Nipponbare and C331 for 48 h. (**C**) Weight gain of newly emerged female adult BPHs fed on the Nipponbare and C331 for 48 h. (**D**) Results of a two-host choice test for BPH insects on the Nipponbare and C331 rice plants. The experiments were repeated with 20 BPH insects per variety (**B**,**C**). Fifteen BPH insects were placed on each pair of rice line and 6 replicates were used for analysis (**D**). Error bars, mean ± SE (**B**–**D**). Asterisks indicate significant differences revealed by Student’s *t* test, at * *p* < 0.05 and ** *p* < 0.01, respectively.

This study evaluated the resistance of rice varieties Nipponbare and C331 to BPH at the seeding stage. Nipponbare was susceptible, and the plants died on the 5th day after BPH infestation. C331 plants grew normally under BPH infestation and showed strong resistance (Figure 1A). To confirm these results, we fed the newly emerged female BPHs on the Nipponbare and C331 plants for 48 h and investigated the honeydew excretion and BPH body weight. The honeydew excretion and body gain were significantly lower for BPHs fed on C331 plants than those on Nipponbare plants (Figure 1B,C and Table S1), suggesting an antibiotic effect of C331. Furthermore, in the two-host choice test, the number of BPHs hat settled on the C331 plants was significantly lower than that on the Nipponbare plants from 2 h to 72 h after BPH was released (Figure 1D and Table S1). These results demonstrated that Nipponbare was susceptible, but C331 was resistant to BPH.

### 2.2. Rice Metabolome Profiling in Response to BPH Feeding

**Figure 2:**
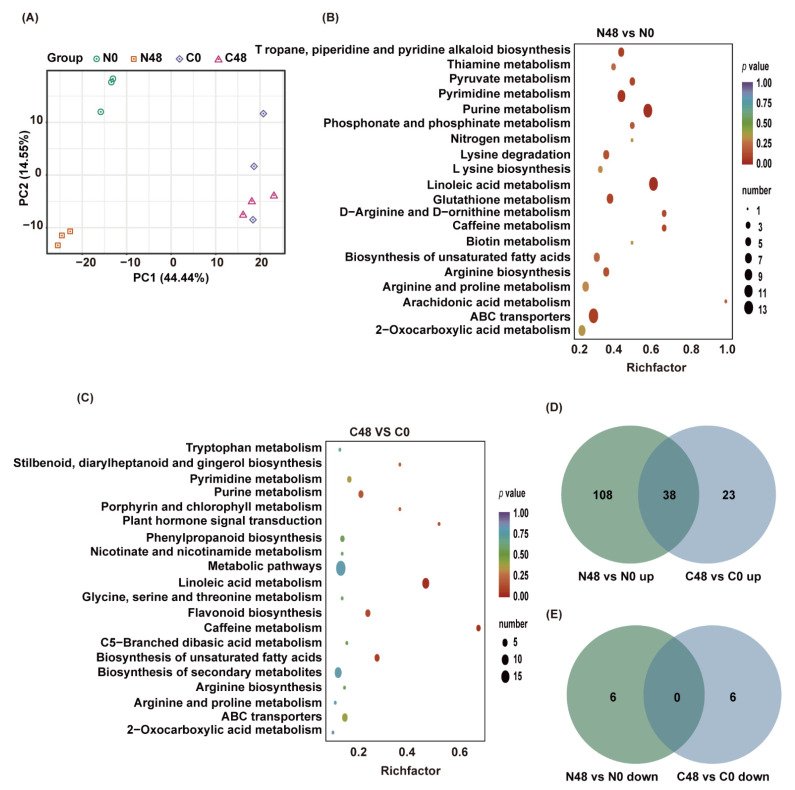
Different accumulated metabolites (DAMs) of Nipponbare and C331 fed by BPH for 48 h. (**A**) PCA plots of metabolism identified by LC-MS/MS of Nipponbare and C331 attacked by BPH at 0 and 48 h post-infestation. (**B**) Enrichment scatter diagram of the top 20 KEGG pathways of DAMs in Nipponbare exposed to BPH stress for 48 h. (**C**) Enrichment scatter diagram of the top 20 KEGG pathways of DAMs in C331 exposed to BPH stress for 48 h. (**D**) Venn diagram of upregulated DAMs in C331 and Nipponbare. (**E**) Venn diagram of downregulated DAMs in C331 and Nipponbare.

To explore the metabolic responses of the resistant and susceptible rice plants to BPH insects, metabolites were extracted from leaf sheaths of rice plants fed on by BPH for 48 h (designated as N48 for Nipponbare, C48 for C331) and the unfed plants (N0, C0), and analyzed using UPLC-MS/MS. A total of 945 metabolites were detected, which belonged to 11 classes (Table S2). Principal component analysis (PCA) showed a significant difference among the different treatments, and the three biological repeats in the same treatment were clustered together (Figure 2A).

We detected 152 differentially accumulated metabolites (DAMs) with |log2FC| ≥ 1 and VIP ≥ 1 between N48 and N0 in BPH-susceptible rice Nipponbare, among which 146 were upregulated and 6 were downregulated in the BPH-fed plants (N48) compared with the unfed plants (N0) (Table S3). In resistant rice C331, there were 67 DAMs, much fewer than those in Nipponbare, among which 61 were upregulated and 6 were downregulated in C48 compared with C0 (Table S4).

We performed a KEGG functional analysis to explore the functions of the metabolites that altered in rice plants after BPH infestation. We found that DAMs in N48/N0 were significantly enriched in many pathways, such as linoleic acid metabolism, purine metabolism, pyrimidine metabolism, ABC transporters, Tropane, piperidine and pyridine alkaloid biosynthesis (Figure 2B and Table S5). In contrast, most DAMs in C48/C0 were significantly enriched in linoleic acid metabolism, caffeine metabolism, biosynthesis of unsaturated fatty acids, flavonoid biosynthesis, and plant hormone signal transduction (Figure 2C and Table S6). These findings indicated that some primary metabolisms were significantly affected in both Nipponbare and C331, but some secondary metabolisms were only significantly affected in the C331 variety.

To further investigate the similarities and differences between Nipponbare and C331 to BPH at the metabolic level, we performed a comparative analysis of their differential metabolites. The results revealed 38 compounds that overlapped between N48/N0 and C48/C0, and these 38 compounds were all upregulated in both rice varieties (Figure 2D), including 27 lipids, 5 nucleotides and derivatives, 5 alkaloids, and 1 phenolic acid (Table S7). Corresponding to that, in the Nipponbare variety, 108 and 6 DAMs were specifically up- and downregulated, respectively, after BPH feeding; while, in the C331 variety, 23 and 6 DAMs were specifically up-and downregulated, respectively (Figure 2D,E), which indicated that these unique DAMs might be responsible for their resistance or susceptibility to BPH.

To further highlight these differences and reveal central tendencies, we draw heat maps for these 143 unique DAMS to show the differences in both rice varieties. We found these DAMs belong to nine classes: alkaloids, amino acids and derivatives, flavonoids, lignans and coumarins, lipids, nucleotides and derivatives, organic acids, others, and phenolic acids (Table S8). Amino acids and derivatives, nucleotides and derivatives, lipids, and most organic acids belong to energy and primary metabolic pathways. Most of these DAMs showed a sharper uptrend in Nipponbare than in the C331 variety (Figure S1 and Table S8). Alkaloids, flavonoids, phenolic acids, lignans and coumarins belong to secondary metabolism. The trend of these DAMs was not distinct. The most notable DAMs were flavonoids, which were downregulated in Nipponbare but upregulated in C331 (Figure S1 and Table S8).

Taken together, these results demonstrated that BPH had a profound impact on Nipponbare, and a more modest effect on C331. The most notable DAMs were flavonoids, which were downregulated in Nipponbare but significantly upregulated in C331.

### 2.3. Transcriptome Analysis of Rice Responses to BPH Feeding

**Figure 3:**
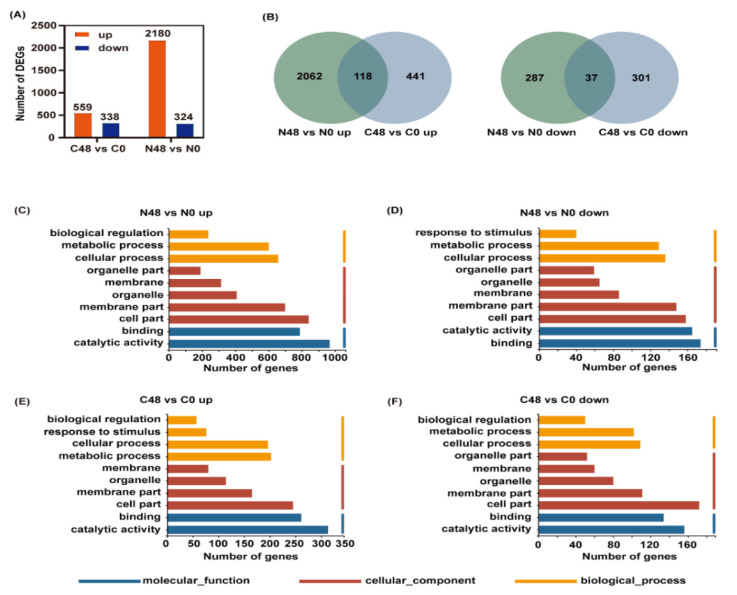
Differentially expressed genes (DEGs) and enriched GO terms analysis in Nipponbare and C331 variety fed by BPH for 48 h compared with untreated control. (**A**) Number of up- and downregulated DEGs in Nipponbare and C331 variety after BPH feeding. (**B**) Venn diagram of up- and downregulated DEGs in Nipponbare and C331 variety fed by BPH for 48 h compared with untreated control. (**C**) Enriched GO terms of upregulated DEGs in Nipponbare fed by BPH for 48 h (N48 vs. N0 up). (**D**) Enriched GO terms of downregulated DEGs in Nipponbare fed by BPH for 48 h (N48 vs. N0 down). (**E**) Enriched GO terms of upregulated DEGs in C331 fed by BPH for 48 h (C48 vs. C0 up). (**F**) Enriched GO terms of downregulated DEGs in C331 fed by BPH for 48 h (C48 vs. C0 down).

To further investigate the molecular effects of BPH on rice varieties, we also performed a transcriptomic analysis. Adjust *p*-value ≤ 0.05 and |log2 Fold Change| ≥ 1 were set as the thresholds to determine the significance of the difference in gene expression between samples. In the Nipponbare variety after BPH feeding, 2504 DEGs, including 2180 that were upregulated and 324 that were downregulated, were identified, whereas only 897 DEGs, including 559 that were upregulated and 338 that were downregulated, were identified in the C331 variety (Figure 3A and Tables S9 and S10). Similarly, there were more DEGs in Nipponbare than C331. These showed gene expression levels for the difference, under BPH infestation, between Nipponbare and C331.

To identify the possible BPH-resistance-related genes, Venn diagrams were used to analyze the up- and downregulated DEGs of the two rice varieties. In the Nipponbare variety, 2062 and 287 DEGs were specifically up- and downregulated, respectively, after BPH feeding; while, in the C331 variety, 441 and 301 DEGs were specifically up- and downregulated, respectively (Figure 3B), which indicated that these unique DEGs might be responsible for their resistance or susceptibility to BPH.

GO enrichment analysis was used to understand the function of these DEGs. The up-and downregulated DEGs in the Nipponbare and C331 plant after BPH feeding were both enriched in the same GO term of biology process (BP), cellular component (CC), and molecular function (MF), such as cellular process, metabolic process, cell part, membrane part, catalytic activity and binding (Figure 3C–F). However, the DEGs that were enriched in terms of their response to stimulus had the opposite response, and were downregulated in the Nipponbare and upregulated in the C331 (Figure 3D,E).

To better understand the function of these DEGs in the Nipponbare and C331 varieties, we performed a Mapman analysis of the DEGs involved in the biotic stress. The result showed that the DEGs involved in MAPK, hormone signaling, transcription factors, cell wall and secondary metabolites were significantly changed in both rice varieties. Still, the changing trend and number of DEGs differed for the two rice varieties (Figure S2A,B). For example, the DEGs related to salicylic acid (SA) were upregulated in the C331 variety but were downregulated in the Nipponbare, while DEGs related to auxins were downregulated in the C331 variety but upregulated in the Nipponbare (Figure S2A,B). Moreover, the number of DEGs related to secondary metabolites in the Niponbare was higher than that in the C331.

Taken together, these results indicated that, when rice was attacked by brown planthopper, most of the changed physiological activities of resistant and susceptible varieties were similar; but the level of trend and variation differed between the two varieties. The response of Nipponbare to BPH was more sensitive and intense than the C331 variety.

### 2.4. Combined Transcriptomic and Metabolomic Analysis

**Figure 4:**
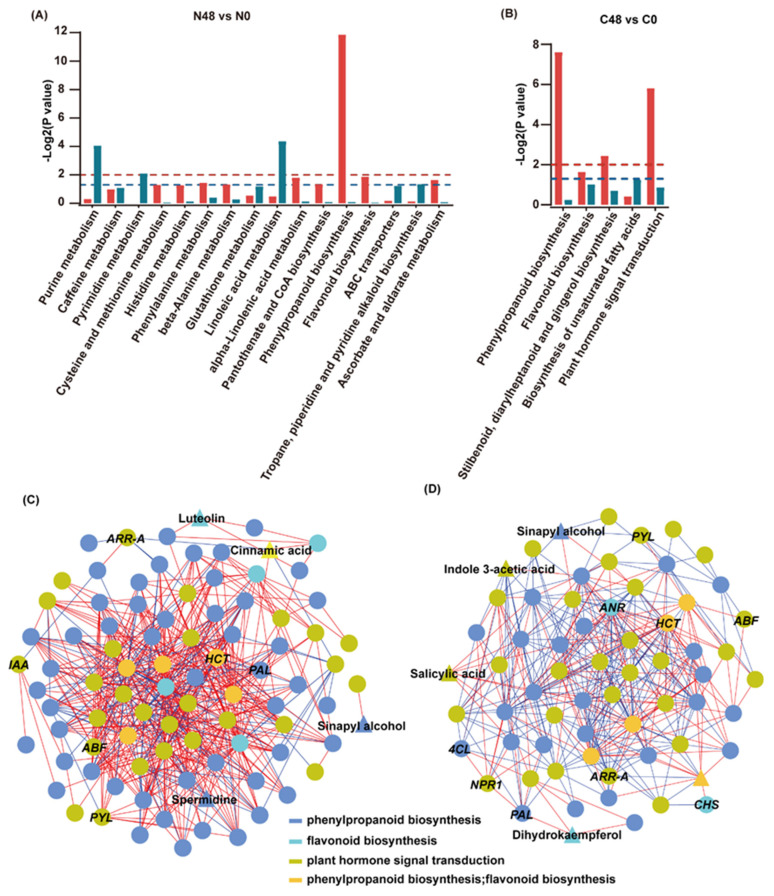
Correlation analysis of the transcriptomic and metabolomic data for Nipponbare and C331 under BPH stress. (**A**) KEGG enrichment analysis of the DEGs (red column) and DAMs (dark cyan column) that were enriched in the same pathway in the Nipponbare. (**B**) KEGG enrichment analysis of the DEGs (red column) and DAMs (dark cyan column) that were enriched in the same pathway in the C331. The red dashed lines represent empirical -Log2P values of 0.01, while the blue dashed lines represent empirical -Log2P values of 0.05. (**C**) The connection network between DEGs and DAMs mapped on phenylpropanoid biosynthesis, flavonoid biosynthesis and plant hormone signal transduction with the absolute value of |pearson correlation coefficient| ≥ 0.9 in the Nipponbare variety. (**D**) The connection network between DEGs and DAMs mapped on phenylpropanoid biosynthesis, flavonoid biosynthesis and plant hormone signal transduction with the absolute value of |pearson correlation coefficient| ≥ 0.9 in the C331 variety. Circle represents DEGs and triangle represents DAMs. Circles and triangles in different color indicates different pathways, dark blue represents phenylpropanoid biosynthesis, bright green represents flavonoid biosynthesis, grass green represents plant hormone signal transduction, yellow–orange represents both phenylpropanoid biosynthesis and flavonoid biosynthesis. The red line represents a positive correlation; the blue line represents a negative correlation.

To further understand the regulatory network of rice in response to BPH infestation, we compared the enrichment of DEGs and DAMs in the Nipponbare and C331 varieties. In the Nipponbare, DEGs and DAMs were significantly enriched in linoleic acid metabolism, purine metabolism, pyrimidine metabolism, phenylpropane biosynthesis and flavonoid biosynthesis (Figure 4A). However, the DEGs and DAMs in the C331 were significantly enriched in terms of phenylpropane biosynthesis, flavonoid biosynthesis, stilbenoid, diarylheptanoid and gingerol biosynthesis and plant hormone signal transduction (Figure 4B). These results suggested that both primary metabolism and secondary metabolism were significantly affected in Nipponbare, but only secondary metabolism was significantly affected in C331. The plant hormone signal transduction pathway was only significantly enriched in C331, and this indicated that it played an important role against BPH in the resistant variety. In addition, although the DEGs and DAMs were enriched in the phenylpropane biosynthesis and flavonoid biosynthesis in both the Nipponbare and C331, the DEGs and DAMs enriched in the flavonoid biosynthesis were all upregulated in the C331, but some were downregulated in the Nipponbare (Table S11). Although the DAMs and most DEGs enriched in the phenylpropane biosynthesis were upregulated in the Nipponbare and C331 varieties, the genes differed (Table S11). The results suggested that flavonoid biosynthesis and phenylpropane biosynthesis played a different role during BPH infestation in the Nipponbare and C331 (Table S11).

To better understand these three rice pathways against BPH, we conducted a correlation analysis of genes and metabolites mapped on these three pathways in Nippon-bare and C331 varieties. First, we calculated the correlation between metabolite content and gene expression in these two rice varieties. Then, we constructed a network map of gene-metabolites based on the Spearman correlation coefficient (with correlation coefficient >0.9 or <−0.9) using Cytoscape. We found strong correlations between genes and genes, genes and metabolites, metabolites and metabolites, in these three pathways in the Nipponbare and C331 varieties (Figure 4C,D and Tables S12 and S13). These results demonstrated an interdependence among plant hormone signal transduction, phenylpropane biosynthesis and flavonoid biosynthesis in rice defense to BPH.

### 2.5. Effects of Plant Hormone on Rice Resistance against BPH

Transcriptome analysis and combined analysis showed that plant hormone plays a vital role in rice against BPH. There were 27 DEGs in Nipponbare and 28 DEGs in C331 involved in plant hormone signal transduction, respectively (Figure S3A,B). *ABF* (ABRE binding factor) and *PYL* (pyrabactin resistance 1-like protein) in the abscisic acid (ABA) signaling pathway were significantly upregulated, *ARR-A* (Type-A response regulator) in the cytokinin (CK) signaling pathway and phytochrome interacting factor 4 (*PIF4*) in the gibberellin (GA) signaling pathway were significantly downregulated in both varieties (Figure S3A,B). However, the non-expressor of pathogenesis-related genes 1 (*NPR1),* an essential regulator of SA, was only upregulated in the C331, and the DEGs involved in auxin signaling were upregulated in the Nipponbare and downregulated in the C331 (Figure S3A,B).

**Figure 5:**
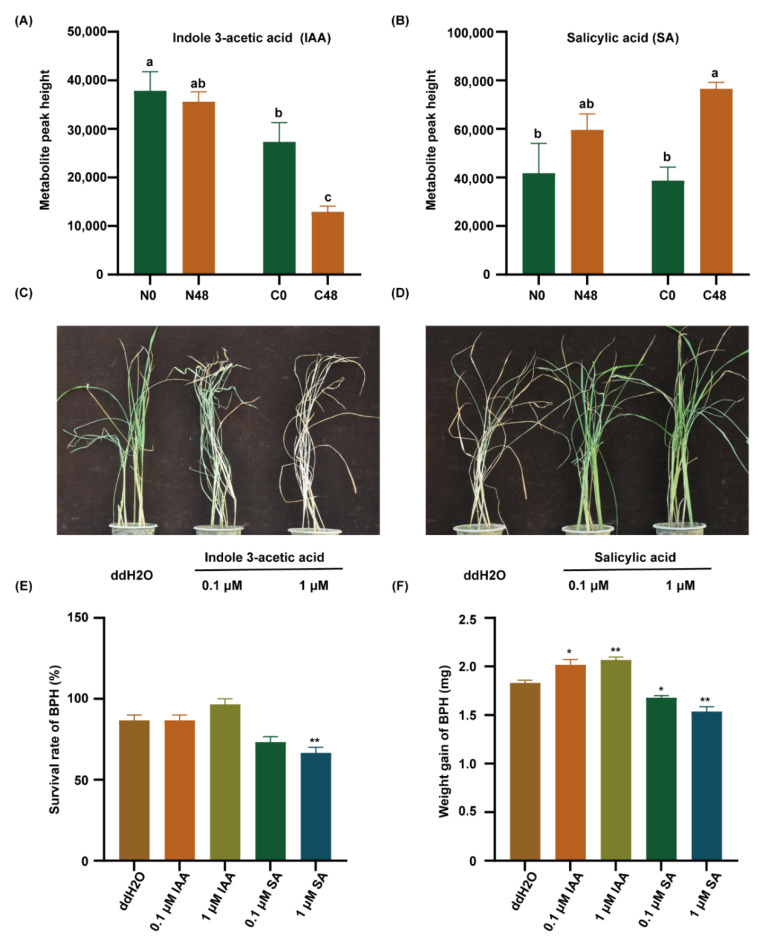
Indole 3-acetic acid (IAA) and salicylic acid (SA) regulate BPH resistance in rice. (**A**) The content of IAA in rice leaf sheath. (**B**) The content of SA in rice leaf sheath. Different lowercase letters above the bars indicate significant differences (*p* < 0.05 by one-way ANOVA with LSD’s post-hoc test), all data were presented as the mean value ± SE(A,B). (**C**) A representative image of the Nipponbare plants after pre-treatment with ddH2O, 0.1 µM IAA, or 1 µM IAA applied to plant leaf sheaths for 2 h followed by 10 third-instar BPH nymphs per plant for 6 days. (**D**) A representative image of the Nipponbare plants after pre-treatment with ddH2O, 0.1 µM SA, or 1 µM SA applied to plant leaf sheaths for 2 h followed by 10 third-instar BPH nymphs per plant infestation for 8 days. (**E**) The survival rate of BPH fed on Nipponbare plants pre-treatment with ddH2O, 0.1 μM IAA, 1 μM IAA, 0.1 µM SA, or 1 µM SA applied to 1/2 MS for 7 days followed by female BPH adult infestation for 48 h. (**F**) Statistical analyses of weight gain of BPH fed on Nipponbare plants pre-treatment with ddH2O, 0.1-μM IAA, 1 μM IAA, 0.1 µM SA, or 1 µM SA applied to 1/2 MS for 7 days followed by female BPH adult infestation for 48 h. Three replicates were used for analysis (**A**,**B**,**E**,**F**). The asterisk represented a significant difference between the treatment and the control—*, *p* < 0.05; **, *p* < 0.01 by one-way ANOVA with Dunnett’s post-hoc test (**E**,**F**). All data were presented as the mean value ± SE.

Therefore, we detected the IAA and SA content in both varieties before and after BPH feeding. The content of IAA was significantly lower in the C331 variety than that in the Nipponbare after BPH feeding (Figure 5A). However, the content of SA was significantly higher in the C331 (Figure 5B). These results suggested that SA might play a positive role in the C331 against BPH, but IAA did the opposite. Then, to confirm the role of IAA and SA in rice against BPH, exogenous IAA and SA were applied to treat the seedlings of Nipponbare to investigate rice’s response to BPH. After BPH feeding for six days, when the Nipponbare plants treated with IAA began to wither and die, the untreated Nipponbare grew well (Figure 5C). After BPH feeding for eight days, when the untreated Nipponbare died, the Nipponbare plants treated with SA grew well (Figure 5D).

In addition, exogenous IAA was also applied to treat the seedlings of C331 to investigate the response of rice to BPH. After BPH feeding for four days, the leaves of C331 plants treated with IAA began to curl, the untreated C331 grew well (Figure S4A). We then examined the performance of rice that were pretreated with auxin transport inhibitor 2,3,5-triiodobenzoic acid (TIBA) and found that the resistance of Nipponbare to BPH increased (Figure S4B).

We also cultured Nipponbare in 1/2 MS supplemented with IAA or SA, respectively, and released the BPHs to feed on these plants for 48 h, and then calculated the survival rate and weight gain in BPH. The results showed that the survival rate of BPHs feeding on the Nipponbare supplemented with SA was lower than that fed on the Nipponbare with IAA (Figure 5E). Similarly, the weight gain of BPHs feeding on the Nipponbare supplemented with SA was lower than those feeding on the Nipponbare with IAA (Figure 5F). These results indicated that IAA decreased, but SA increased rice resistance to BPH.

### 2.6. Phenylpropane Metabolism Was Induced by Brown Planthopper Feeding

**Figure 6:**
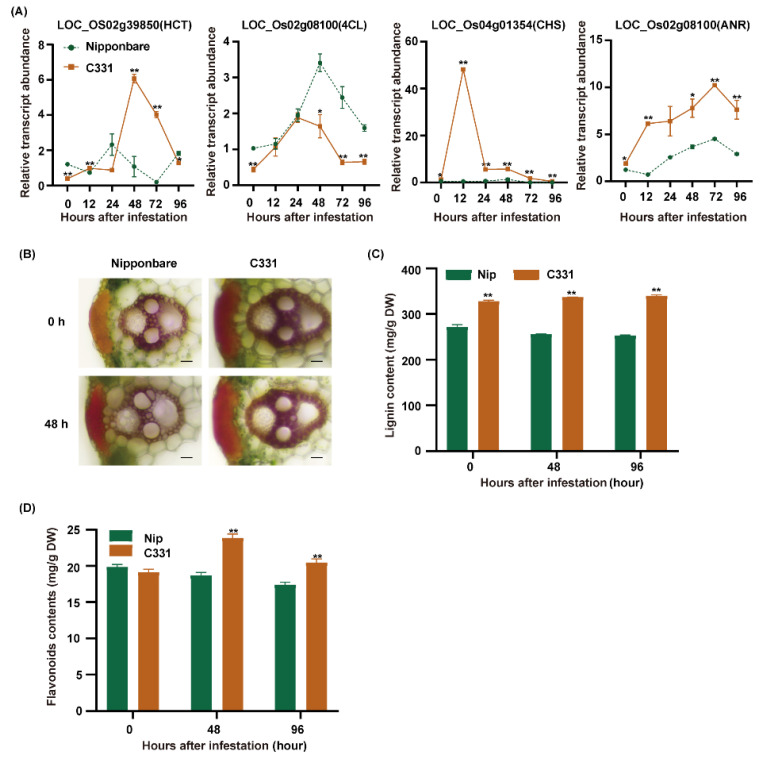
The gene expression and metabolites changes in the network of phenylpropanoid metabolism induced by BPH infestation. (**A**) Quantitative real-time PCR analyses of lignin and flavonoids biosynthesis pathway genes in Nipponbare and C331 attacked by BPH for 0 h, 12 h, 24 h, 48 h,72 h and 96 h. (**B**) Histochemical staining showing lignin accumulation in leaf sheaths fed by BPH for 0 h and 48 h (scale bars, 20 μm). (**C**) Lignin contents of Nipponbare and the C331 plants fed by BPH for 0 h, 48 h and 96 h, measured using the acetyl bromide method. (**D**) Flavonoids contents of Nipponbare and the C331 plants fed by BPH for 0 h, 48 h and 96 h—*, *p* < 0.05; **, *p* < 0.01 by one-way ANOVA with Dunnett’s post-hoc test (**C**,**D**). All data are presented as the mean value ± SE.

The combined analyses suggested that phenylpropane biosynthesis and flavonoid biosynthesis, which are two major branches of phenylpropanoid metabolism, played important roles in rice against BPH [28]. First, we found many markedly regulated genes related to lignin and flavonoid biosynthesis (Figure S5). There were four DEGs, including *HCT* (hydroxycinnamoyl-CoA shikimate/quinate hydroxycinnamoyl transferase), *4CL* (4-coumarate-CoA ligase), *CHS* (chalcone synthase) and *ANR* (anthocyanidin reductase) were only upregulated in the C331. Then, we detected the expression of these genes from 0 to 96 h after BPH infestation in the Nipponbare and C331, and these four genes were significantly upregulated in the C331 (Figure 6A). The results suggested that lignin and flavonoids might play an important role in rice against BPH.

Phenylpropane biosynthesis can give rise to lignin by HCT and a series of enzymes, and flavonoid biosynthesis can give rise to flavonoids such as chalcones, leucoanthocyanidins and anthocyanidins by CHS and a series of enzymes [28]. To verify the role of lignin and flavonoids in rice against BPH, we measured the lignin and flavonoids contents of the Nipponbare and C331 varieties. First, we examined the lignin accumulation in leaf sheaths stained with phloroglucinol and measured the lignin content of these varieties. We found that sclerenchyma in the C331 was thicker than in the Nipponbare, and the lignin content of the C331 was higher than in the Nipponbare before and after BPH feeding (Figure 6B,C). Then, we measured the flavonoids of Nipponbare and C331 and found a significant rise in flavonoids in the C331 but a decrease in the Nipponbare after BPH feeding (Figure 6D).

In addition, we evaluated the BPH resistance and measured the lignin and flavonoids contents in another three rice varieties. The results showed that the weight gain for BPHs fed on ZH11 was higher than those fed on the C700 and OFF, which indicated that ZH11 was susceptible to BPH but C700 and OFF were resistant to BPH (Figure S6A). The contents of lignin and flavonoids in the C700 and wild rice were higher than those in the ZH11 (Figure S6B,C). Finally, we overexpressed CHS (LOC_Os04g01354) and HCT (LOC_Os02g39850) genes into rice protoplasts to measure the flavonoids content. The results showed that the flavonoids content of the rice protoplasts that overexpressed CHS genes was higher, and the survival rate of BPH fed with the extract from the rice protoplasts with an overexpressed CHS gene was lower (Figure S7A,B).

These results suggested that lignin and flavonoids play an important role against BPH in rice.

### 2.7. Epigallocatechin Positively Regulated Rice Resistance to BPH

**Figure 7:**
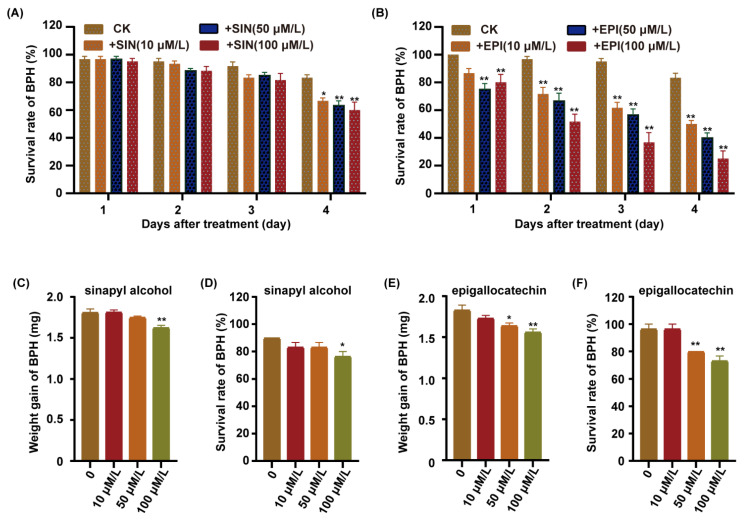
Functional validation of candidate metabolites. (**A**) Survival rate of BPH fed on artificial diet supplemented with different concentrations of sinapyl alcohol. (**B**) Survival rate of BPH fed on artificial diet supplemented with different concentrations of epigallocatechin. (**C**) Weight gain of BPH fed on Nipponbare plants pre-treatment with sinapyl alcohol applied to 1/2 MS at concentrations of 0 μM/L, 10 μM/L, 50 μM/L or 100 μM/L for 7 days, followed by female BPH adult infestation for 48 h. (**D**) Survival rate of BPH fed on Nipponbare plants pre-treatment with sinapyl alcohol applied to 1/2 MS at concentrations of 0 μM/L, 10 μM/L, 50 μM/L or 100 μM/L for 7 days, followed by female BPH adult infestation for 48 h. (**E**) Weight gain of BPH fed on Nipponbare plants pre-treatment with epigallocatechin applied to 1/2 MS at concentrations of 0 μM/L, 10 μM/L, 50 μM/L or 100 μM/L for 7 days, followed by female BPH adult infestation for 48 h. (**F**) Survival rate of BPH fed on Nipponbare plants pre-treatment with epigallocatechin applied to 1/2 MS at concentrations of 0 μM/L, 10 μM/L, 50 μM/L or 100 μM/L for 7 days, followed by female BPH adult infestation for 48 h. Error bars, mean ± SE of 6 biological replicates (**A**,**B**) or three replicates (**C**–**F**), The asterisk represent a significant difference between the treatment and the control. *, *p* < 0.05; **, *p* < 0.01 by one-way ANOVA with Dunnett’s post-hoc test. All data are presented as the mean value ± SE.

To further investigate which metabolites were involved in rice against BPH, we integrated the transcriptomic and metabolomic analyses of the lignin and flavonoid biosynthesis pathway. We found that the sinapyl alcohol content was upregulated in the lignin biosynthesis pathway, and the expression of the ANR gene was upregulated in the flavonoid biosynthesis pathway in the C331 after BPH feeding (Figure 6A and Figure S4). P-hydroxyphenyl (H), guaiacyl (G), and syringyl (S) are three natural lignin polymers and can be generated from monolignols, p-coumaryl alcohols, coniferyl alcohols, and sinapyl alcohols, respectively [28]. A previous study showed that the anthocyanidin reductase encoded by ANR catalyzed (−)-Epi-flavan-3-ols into the epigallocatechin [28]. We fed the BPH with an artificial diet, supplemented with different sinapyl alcohol and epigallocatechin content, for four days, respectively. The survival rate of BPHs did not decrease until four days after feeding the sinapyl alcohol content (Figure 7A). In contrast, the survival rate of BPHs showed a rapid decrease compared with the control after feeding with epigallocatechin content (Figure 7B). As the concentration of the epigallocatechin increased, the survival rate decreased more rapidly.

We also cultured Nipponbare in 1/2 MS supplemented with sinapyl alcohol and epigallocatechin, respectively, released the BPHs to feed on these plants for 48 h, and then calculated the weight gain and survival rate for the BPHs. The results showed that the weight gain and survival rate of BPHs fed on the Nipponbare supplemented with the sinapyl alcohol was minimal, and only lower at the 100 μM/L concentration (Figure 7C,D). However, the weight gain and survival rate of BPHs fed on the Nipponbare supplemented with the epigallocatechin was significantly lower than the control, and the higher the concentration of the epigallocatechi, the lower the weight gain and survival rate of BPH (Figure 7E,F). These results indicated that the epigallocatechin had significant lethal effects on BPH, but the sinapyl alcohol did not.

## 3. Discussion

Rice is one of the major cereal crops, feeding about three billion people globally, and is often attacked by various insect pests [2]. Host plant resistance of rice is an effective and environmentally sustainable approach to reduce insect damage [3]. Previous studies primarily focused on the localization and isolation of insect resistance genes [4,5,7,8,10], paying less attention to the metabolic and transcriptomic mechanism. We integrated the metabolomic and transcriptomic analysis to reveal that IAA negatively regulated resistance to BPH, and epigallocatechin enhanced resistance to BPH.

To understand the molecular mechanism of rice resistance to BPH, the metabolome and transcriptome were performed in the Nipponbare and C331 plants under BPH infestation. A total of 945 metabolites were detected in this study, including 152 DAMs in the Nipponbare and 67 DAMs in the C331 plants. Interestingly, the DAMs in the Nipponbare were mostly enriched in the primary metabolism, such as purine metabolism and pyrimidine metabolism, which showed a perturbation of energy metabolism in the susceptible rice Niponbare. Most DAMs in Nipponbare belong to lipids, nucleotides and derivatives, amino acids and derivatives. However, the DAMs in the C331 were mainly enriched in the secondary metabolism, and these DAMs included alkaloids, phenolic acids, flavonoids, lignans and coumarins. These results suggested that the lipid, nucleotides, and amino acids must be synthesized to compensate for the nutrient loss by BPH feeding on the susceptible rice, while the secondary metabolites, including flavonoids and lignans, were produced to prevent BPH feeding in the resistant rice. Transcriptome analysis revealed significant differences in the response of the Nipponbare and C331 plants following BPH infestation. The responses to BPH in the Nipponbare plants were more robust than the C331, as higher number of DEGs were detected in the Nipponbare plants, which indicated significant physiological and gene expression changes in the Nipponbare plants after BPH feeding. In addition, the upregulated DEGs were much higher than the downregulated ones in the C331 plants, suggesting that the upregulated expressions of genes enhanced rice resistance to BPH. Susceptible rice was more sensitive to BPH than resistant rice, as more DAMs and DEGs were identified in susceptible rice than resistant rice. BPH infestation affected all metabolism levels, disrupting energy supply in the susceptible rice. While primary metabolism was mildly changed and some secondary metabolisms were significantly induced in resistant rice, these indicated that secondary metabolisms played an important role in rice resistance to BPH. In addition, DAMs belonging to flavonoids and DEGs related to stimulus were upregulated in resistant rice, indicating that flavonoids and the related genes played a significant role in the rice defense response to BPH infestation. Finally, we integrated the transcriptome and metabolome analysis to find that plant hormone signaling transduction, phenylpropane biosynthesis, and flavonoid biosynthesis were involved in rice resistance against BPH.

Plant hormones play important roles in plant against insects and pathogens [29,30,31,32,33,34]. Previous studies had shown that SA is a central hormone in plant defense against pathogens, which regulates both local disease resistance mechanisms and systemic acquired resistance (SAR) [35,36]. Auxin, a crucial role in plant growth and development, is regulated by pathogen infestation [37]. In this study, the higher content of SA was verified in the C331 plants after BPH feeding, and treatment with exogenous SA can enhance rice resistance to BPH (Figure 5D). We also found that the content of IAA and the expression of the genes related to auxin signal transduction in C331 was significantly lower than that of Nipponbare, and the exogenous application of IAA can reduce rice resistance to BPH (Figure 5C). These results suggested that SA and IAA might have antagonistic effects on rice resistance to BPH. Some evidence indicates that SA could suppress auxin signaling to inhibit pathogen infestation [38]. Some studies found that auxin could suppress PR1 expression and SA-dependent defense [37,39]. The antagonistic interactions between SA and auxin signaling possibly occurred because plants need to maintain a balance between growth and defence.

Lignin provided structural support for land plants and limited the invasion of pathogens and insects [40,41,42]. Changing lignin composition caused altered responsiveness to bacterial inoculation in A. thaliana [41]. The enzyme encoded by HCT catalyzed the p-Coumarocyl-CoA into the lignin biosynthesis pathway (Figure S4); HCT-silenced plants exhibited an adverse lignin content and structure change in the cell wall, leading to increased susceptibility to degradation by cell-wall-degrading enzymes secreted by pathogens [43]. In this study, the thickness of the sclerenchyma tissue and vascular bundle and the total lignin content in the C331 plants were higher than in the Nipponbare (Figure 6B,C). The expression of *HCT* and *4CL* were significantly upregulated, and the sinapyl alcohol in C331 increased with the time of BPH feeding. The survival rate of BPH did not decrease until four days after feeding the sinapyl alcohol. These results suggested that lignin accumulation in the C331 plants was constitutively higher, and lignin served as a physical barrier that mainly prevented BPH stylets from penetrating the phloem rather than killing BPH.

Flavonoids also played important roles in the interaction between plants and parasites [44,45,46]. For example, quercetin, kaempferol, and rutin were found toxic to Spodoptera litura larvae [44,45], tricin can also defend the rice plant against infestation by BPH [46]. CHS was the first committed enzyme in flavonoid biosynthesis and can switch from phenylpropanoid metabolism to flavonoid metabolism [28]. Furthermore, increased CHS transcription co-segregated with dicamba resistance and overexpression of CHS gene enhanced HL resistance by synthesizing more anthocyanin [47]. Kumar found that the pyramiding of tea anthocyanidin reductase (ANR) and di-hydroflavonol-4-reductase (DFR) increased flavan-3-ols and improved protective ability under stress conditions in tobacco [48]. In this study, the total content of flavonoids in the C331 plants was higher than in the Nipponbare after BPH feeding, but there was no significant difference before BPH feeding (Figure 6D). The expressions of *CHS* and *ANR* were significantly upregulated in the C331 (Figure 6A and Figure S4). Furthermore, the survival rate of BPH rapidly decreased after feeding the epigallocatechin, which was the substrate of ANR. These results suggested that the accumulation of induced flavonoids in the C331 plants could elicit a direct toxicity effect on BPH. The epigallocatechin was found to reduce aphid survival by decreasing the activity of detoxification enzymes [49].

Plants have evolved complex defense mechanisms against various herbivorous insects over the long period of their evolution. Moreover, plant hormone and secondary metabolites play important roles in biotic and abiotic stress defense [28,50]. In this study, SA played a positive role, and IAA played a negative role in rice resistance to BPH; the constitutive lignin and induced flavonoids also played a determinant role in rice resistance. Therefore, our work provides new insights into plant–insect interactions and may help to improve rice resistance against the insect.

## 4. Materials and Methods

### 4.1. Plants and Insects

Five rice varieties were used in this study. Nipponbare and ZH11 were susceptible rice cultivars; C331 and C700 were resistant varieties obtained from the International Rice Research Institute. OFF was wild-rice Oryza officinalis obtained from the Guangxi Academy of Agricultural Sciences.

Brown planthopper population was maintained on the susceptible cultivar TN1 under controlled environmental conditions ((26 ± 0.5) °C, 16:8 h light:dark photoperiod) as previously described [51].

### 4.2. BPH Resistance Evaluation

Ten seeds of Nipponbare and C331 were sown in a plastic pot (9 cm × 12 cm) covered with nylon mesh. At the five-leaf stage, the rice seedlings were infested with third instar BPH nymphs at 10 nymphs per plant in each plot. The observation was performed every day until susceptible control was dead, then the rice plants were photographed and scored. At least three replicates were used.

### 4.3. Two-Host Choice Test

A two-host choice test was performed to evaluate the host-selection behavior of BPH insects on Nipponbare and C331, following the method of Zheng et al. [51]. The Nipponbare and C331 plants were grown in the plastic cup (9 cm × 12 cm) covered with nylon mesh, and 15 third BPH nymphs were released in each cup. The number of BPH nymphs that settled on each plant was recorded 1, 2, 4, 8, 12, 24, 36, 48, 60, and 72 h after release. A total of six cups were analyzed for each paired group.

### 4.4. Honeydew Excretion and BPH Weight Gain Measurements

Honeydew excretion and BPH weight gain measurements were performed following the method of Zheng et al. [51]. Nipponbare and C331 plants were grown in a plastic plot (22 cm × 22 cm), 20 plants per cup. At the five-leaf stage, a total of 20 female BPH adults previously starved for 2 h were weighed, then the insect was introduced into a pre-weighted parafilm sachet (1.5 cm × 2.5 cm) fixed on the leaf sheath 2–3 cm above the soil. After 48 h of feeding, the surviving insects and the parafilm sachet were weighted again; the change in insects’ weight was recorded as BPH weight gain, and the change in sachets’ weight was recorded as honeydew excretion.

### 4.5. RNA-Seq and Data Analysis

Five-leaf stage seedings of Nipponbare and C331, previously covered with nylon mesh, were attacked by 10 third instar BPH nymphs per seeding for 48 h. The unfed Nipponbare and C331 samples were used as a control. The leaf sheaths of the plants were collected and frozen in liquid nitrogen, and stored at −80 °C until use. Each sample included three replicates with 10 plants per replicate. RNA Extraction from samples using the FastPure Plant Total RNA Isolation Kit and then the qualified RNAs were used for transcriptome sequencing using Illumina platform. The raw data acquired from the sequencing was screened using Sickle and SeqPrep software to obtain clean data, then the reads were aligned to the reference genome (http://rice.plantbiology.msu.edu/index.shtml, accessed on 10 October 2020) using TopHat2. Finally, mapped reads were assembled by StringTie, and transcripts were compared with annotation databases. To measure the expression of assembled transcripts, FPKM values were determined using RSEM. The differential expression analysis was performed using DESeq2, and genes were considered as differentially expressed genes (DEGs) if *p* ≤ 0.05 and fold change (FC) ≥2 or FC ≤0.5.

### 4.6. Metabolomics and Data Analysis

Widely targeted metabolomics was carried out on three independent biological replicates of Nipponbare and C331, treated with BPH for 48 h, and the unfed were used as controls. Three biological replicates per treatment with 10 seedlings per replicate were used, and the samples were freeze-dried by vacuum freeze-dryer (Scientz-100F, Guangzhou, China). The freeze-dried sample was ground using tissuelyser (64L, Jingxin, Shanghai, China) with a zirconia bead for 1 min at 50 Hz. Metabolites were extracted by 100 mg powder samples in 70% aqueous methanol (1.2 mL) and vortex 30 s every 30 min for 6 times in total, before placing the sample in a refrigerator at 4 °C overnight. Following centrifugation at 12,000 rpm for 10 min, the extracts were filtrated (SCAA-104, 0.22 μm pore size; ANPEL, Shanghai, China, http://www.anpel.com.cn/, accessed on 15 November 2019) before UPLC-MS/MS analysis. The UPLC column was Agilent, SB-C18 1.8 um (2.1 mm-×100 mm), the injection volume was 2 μL, the flow rate was 0.4 mL/min, and the column temperature was 40 °C. Water or acetonitrile containing 0.04% acetic acid were used as aqueous and organic mobile phases, respectively. The gradient elution was 5–95% B in 11 min, 95% B at 11–12 min, 95% B at 12–12.1 min, and 5% B at 12.1–14 min. The mass spectrometry conditions included Lin-ear ion trap (LIT) and triple quadrupole (QQQ) scans. Ion spray voltage of positive ion with 5500 V and negative ion with 4500 V scan modes was adopted for mass spectrometry signal acquisition of the sample, GSI was 50 psi, GSII was 60 psi, and CUR was 25 psi, the source temperature was 550 °C. Then, following data evaluation based on the self-built database MWDB (metware database) and the public database of metabolite information (https://www.mz-clound.org/; https://massbank.eu/MassBank/; https://pubchem.ncbi.nlm.nih.gov/, accessed on 11 November 2020), the primary and secondary spectral data of mass spectrometry were qualitatively analyzed by software Analyst 1.6.3 and quantitatively analyzed by MRM. The differentially accumulated metabolites (DAMs) were identified by variable importance in projection (VIP) ≥1 and fold change (FC) ≥2 or FC ≤0.5.

### 4.7. RNA Isolation and qRT–PCR Analysis

For qRT–PCR analysis of genes to BPH response, 5-week-old rice seedlings were individually infested with 10 third instar BPH nymphs per plant, and leaf sheaths were collected after 0, 12, 24, 48, 72, and 96 h for RNA extraction after BPH feeding. Total RNA from each leaf sample was isolated with TRIzol and extracted with phenol-chloroform. Then, the qualified RNAs were used for reverse transcription to synthesize cDNA. Each gene was amplified using gene-specific primers designed using Primer Blast, and the actin gene was used as an internal control. The mean and standard error were determined from three biological replicates, for each of which the PCR was conducted in triplicated technical repeats. The primers used in this study were listed in Table S14.

### 4.8. Hormone Treatments and BPH Performance

To investigate the effects of IAA or SA on rice defenses, 5 Nipponbare seeds were sown in a 9 cm-diameter plastic pot. At the five-leaf stage, the seedlings were sprayed with Indole 3-acetic acid (IAA; 0, 0.1 μM or 1 μM) or Salicylic acid (SA; 0, 0.1 μM or 1 μM), and third-instar BPH nymphs were released on the seedlings 2 h after spraying at the level of 10 insects per plant. Each treatment was performed in triplicate. Plant damage levels were observed daily. We also tested the effects of IAA on C331′ resistance to BPH and the processing was consistent with Nipponbare.

For a BPH resistance analysis of the IAA or SA-treated plants, 10 Nipponbare seeds were sterilized and cultured in 1/2 MS solid medium applied with 0, 0.1 μM IAA, 1 μM IAA, 0.1 μM SA or 1 μM SA, respectively, and then 10 female BPH adults were applied to the seedlings. Each treatment was carried out in triplicate. The weight of BPH was measured, and the survival percentage was recorded.

To investigate the effects of auxin transport inhibitor 2,3,5triiodobenzoic acid (TIBA) on rice defenses, Nipponbare seeds were sown in a 9 cm-diameter plastic pot. At the four-leaf stage, the seedlings were sprayed with auxin transport inhibitor 2,3,5triiodobenzoic acid (TIBA) (TIBA; 0, 0.1 μM or 1 μM) and third-instar BPH nymphs were released on the seedlings 2 h after spraying at the level of 10 insects per plant. Each treatment was performed in triplicate. Plant damage levels were observed daily.

### 4.9. Lignin Analysis

Quantification of lignin content was performed according to the manufacturer’s instructions (Solarbio, Beijing, China). In brief, the leaf sheaths of five-leaf plants attacked by BPH for 0, 48, and 96 h were harvested individually. Leaf-sheaths were dried to a constant weight, pulverized, filtered with a 40-mesh sieve, and 3 mg collected into 1.5 mL microcentrifuge tubes. After the addition of 300 μL of reagents 1 and 12 μL of perchloric acid, the mixture was incubated in a water bath at 80 °C for 40 min. Then, 300 μL of PCIA was added, after centrifugation for 10 min at 8000× *g*, 12 μL of supernatant was taken and 588 μL of glacial acetic acid was added, the absorbance of the supernatant was measured at 280 nm. Error bars indicate SE of 3 biological replicates.

For the cellular observation of lignin, fresh hand-cut specimens were excised from rice leaf sheath at the five-leaf stage, fixed, sliced into 150 μm thickness, soaked in hydrochloric acid (HCl) and stained with a solution of 5% phloroglucinol in 95% ethanol, which stains lignified tissues red. The stained sections were visualized under an Olympus BX51 microscope (Olympus Optical, Tokyo, Japan). At least 15 sections were observed for each cultivar or line.

### 4.10. Flavonoids Content Measurements

Flavonoid content was measured for rice plants according to the manufacturer’s instructions (Solarbio, Beijing, China). In brief, the leaf sheaths of five-leaf plants attacked by BPH for 0, 48, and 96 h were individually harvested. Leaf-sheaths were dried to a constant weight, pulverized, filtered with a 40-mesh sieve, and 100 mg was collected into 1.5 mL microcentrifuge tubes. A total of 1 mL extracting solution (60% ethanol) was added, and the mixture A was extracted by ultrasonic method (ultrasonic power: 300 W) at 60 °C for 30 min. The crude extract was centrifuged at 12,000 rpm for 10 min, and the volume of supernatant was adjusted to 1 mL. Three different reagents were successively added according to the manufacturer’s instructions, then mixed well, boiled in a water bath at 37 °C for 45 min, and centrifuged at 10,000× *g* for 10 min. A total of 200 μL supernatant was used to determine absorbance values at A470 in a 96-well plate. The flavonoid content was represented using a standard rutin curve, made beforehand.

ZH11, C700 and OFF were treated in the same way.

### 4.11. Vector Construction

The coding sequence (CDS) of LOC_OS02g39850(HCT) and LOC_OS04g01354(CHS) was amplified from C331 using gene-specific primers with linker sequences. Then, the CDS was recombined into the binary vector pCXUN with a HA protein tag under the control of the ubiquitin promoter (UBI) [5,7,52], and the recombination reaction was carried out using the Exnase II enzyme mix (Vazyme, Nanjing, China). Next, the constructed plasmids were transformated into *E. coli* to verified by sequencing. The primers used in this study are given in Table S15.

### 4.12. Extraction and Determination of Total Flavonoids from Rice Protoplasts

For extraction of total flavonoids from rice protoplasts analysis, we grew 600 Nipponbare rice seedlings on 1/2 MS medium for 10 days to prepare rice protoplasts of 5 mL [52]. Then, 300 μL rice protoplasts were aliquoted into 2 mL micro-centrifuge tube, three tubes for each gene, 8 ug of plasmid was added to each one of tubes, transformation was performed using the previously described method [5], and the protoplasts were incubated in W5 solution at 25 °C overnight. The following day, samples were centrifuged (5 min, 800× *g*) and the supernatant was discarded, then, 1 mL extracting solution (60% ethanol) was added, and the mixtures were extracted using the ultra-sonic method (ultrasonic power: 300 W) at 60 °C for 30 min. The crude extract was centrifuged at 12,000 rpm for 10 min, and the volume of super-natant for each tube was adjusted to 1 mL in new tubes. Then, 200 μL supernatant was used to determine absorbance values at A470 in a 96-well plate. Three separate experiments for three genes were performed in triplicate (HCT, CHS, GFP).

### 4.13. Effect of Total Flavonoids Extract from Rice Protoplasts on BPH Survival

The rest of the ethanol extract for rice protoplasts in 4.12 for each gene in each tube was evaporated by a rotator RE-2000, high-purity ethanol (10 ul) was added to each tube, and three tubes for each gene were individually combined into a 200 μL conical tube. Next, the extract for three genes was individually added into the artificial diet D-97 to examine its effect on BPH survival at a volume ratio of 1 μL:1000 μL [53]. Then, 200 μL artificial diet with a different extract was placed on the membrane and sandwiched between two layers of Parafilm^®^ M film wrapping on one end of glass tubes (3.0 cm × 15 cm). Finally, 10 second-instar BPH nymphs was introduced in the glass tubes, and the other end of the tube was covered with gauze to enable ventilation. The artificial diet was renewed every day to keep fresh. Five biological replicates were analyzed for each treatment, and survival rates were recorded every day.

### 4.14. Effect of Sinapyl Alcohol and Epigallocatechin on BPH Survival and Weight Gain

Sinapyl alcohol (purity > 95%, Bidepharm, Shanghai, China) was added into the artificial diet D-97 to examine its effect on BPH survival at a concentration of 0, 10, 50 or 100 μM/L [53]. Then, a 200 μL artificial diet with a different concentration of sinapyl alcohol was placed on the membrane and sandwiched between two layers of Parafilm^®^ M film wrapping on one end of glass tubes (3.0 cm × 15 cm). Then, 10 second-instar BPH nymphs were introduced into the glass tubes, and the other end of the tube was covered with gauze to enable ventilation. The artificial diet was renewed every day to keep it fresh. Six biological replicates were analyzed for each treatment, and survival rates were recorded every day. For a BPH resistance analysis of the sinapyl alcohol-treated plants, 10 Nipponbare seeds were sterilized and cultured in 1/2 MS solid medium supplemented with 0, 10 μM/L, 50 μM/L or 100 μM/L sinapyl alcohol, respectively, and then 10 female BPH adults were applied to the seedlings. Each treatment was carried out in triplicate. The weights of BPH before and after being fed on rice for 48 h were measured, and the survival percentage was also recorded.

Epigallocatechin (purity > 90%, shyuanye Phytochemicals Ltd., Shanghai, China) was added to the artificial diet D-97 to examine its effect on BPH survival at a concentration of 0, 10, 50 or 100 μM/L [53]. The subsequent processing is consistent with the sinapyl alcohol. For a BPH resistance analysis of the epigallocatechin-treated plants, 10 Nipponbare seeds were sterilized and cultured in 1/2 MS solid medium supplemented with 0, 10 μM/L, 50 μM/L or 100 μM/L epigallocatechin, respectively, and then 10 female BPH adults were applied to the seedlings. Each treatment was performed in triplicate. The weights of BPH before and after being fed on rice for 48 h were measured, and the survival percentage was also recorded.

### 4.15. Correlation Analysis of Transcriptomic and Metabolomic Data

We performed a correlation analysis of transcriptomic and metabolomic data to explore the correlation between DEGs and DAMs. First, we selected the DEGs and DAMs mapped on plant hormone signal transduction, phenylpropane biosynthesis and flavonoid biosynthesis in Nipponbare and C331 varieties. Then, the Spearman correlation coefficient (SCC) of the DEGs and DAMs was calculated using the cor function in the R package. Finally, we constructed a network map of gene-metabolite based on the Spearman correlation co-efficient (with correlation coefficient >0.9 or <−0.9) using Cytoscape.

## Data Availability

Not applicable.

## Supplementary Materials

The following supporting information can be downloaded at: https://www.mdpi.com/article/10.3390/ijms23084083/s1.
