# Supplementary material for: Transcriptome and Metabolome Profiling Reveal the Resistance Mechanisms of Rice against Brown Planthopper
Source: Int J Mol Sci. 2022 Apr 7;23(8):4083. doi: 10.3390/ijms23084083 (PMC9031479; doi:10.3390/ijms23084083)
Supplement: Supplementary file 1 [file ijms-23-04083-s001.zip › ijms-supplmentary figure.pdf]

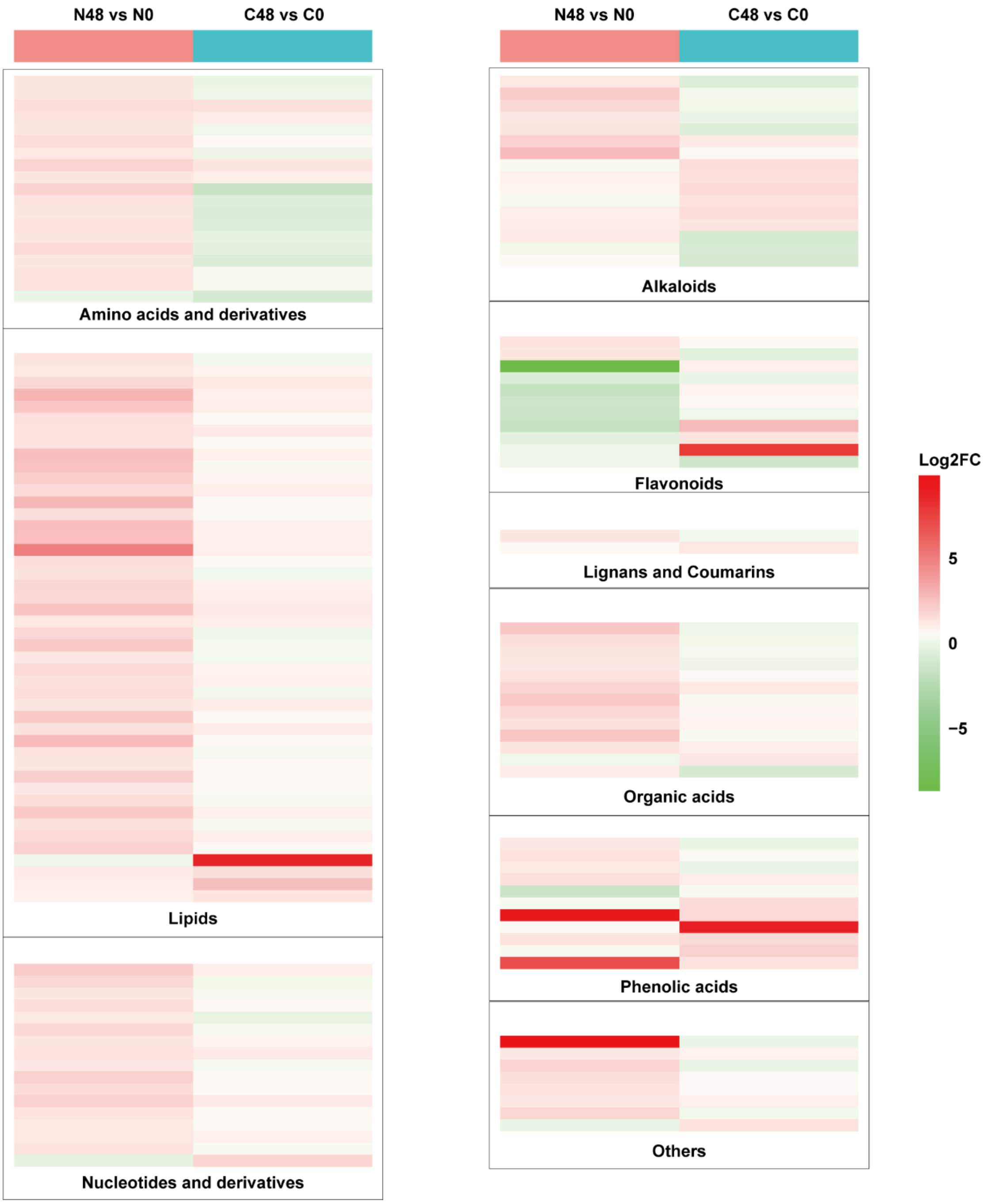

Figure S1. Heatmaps of 143 unique differently accumulated metabolites (DAMs) of Nipponbare and C331 attacked by BPH at 0 and 48 h post-infestation. Heatmap represents the log2-transformed fold changes of DAMs, red circles represent upregulated metabolites, and green circles represent downregulated metabolites. The darker the color, the larger the difference.

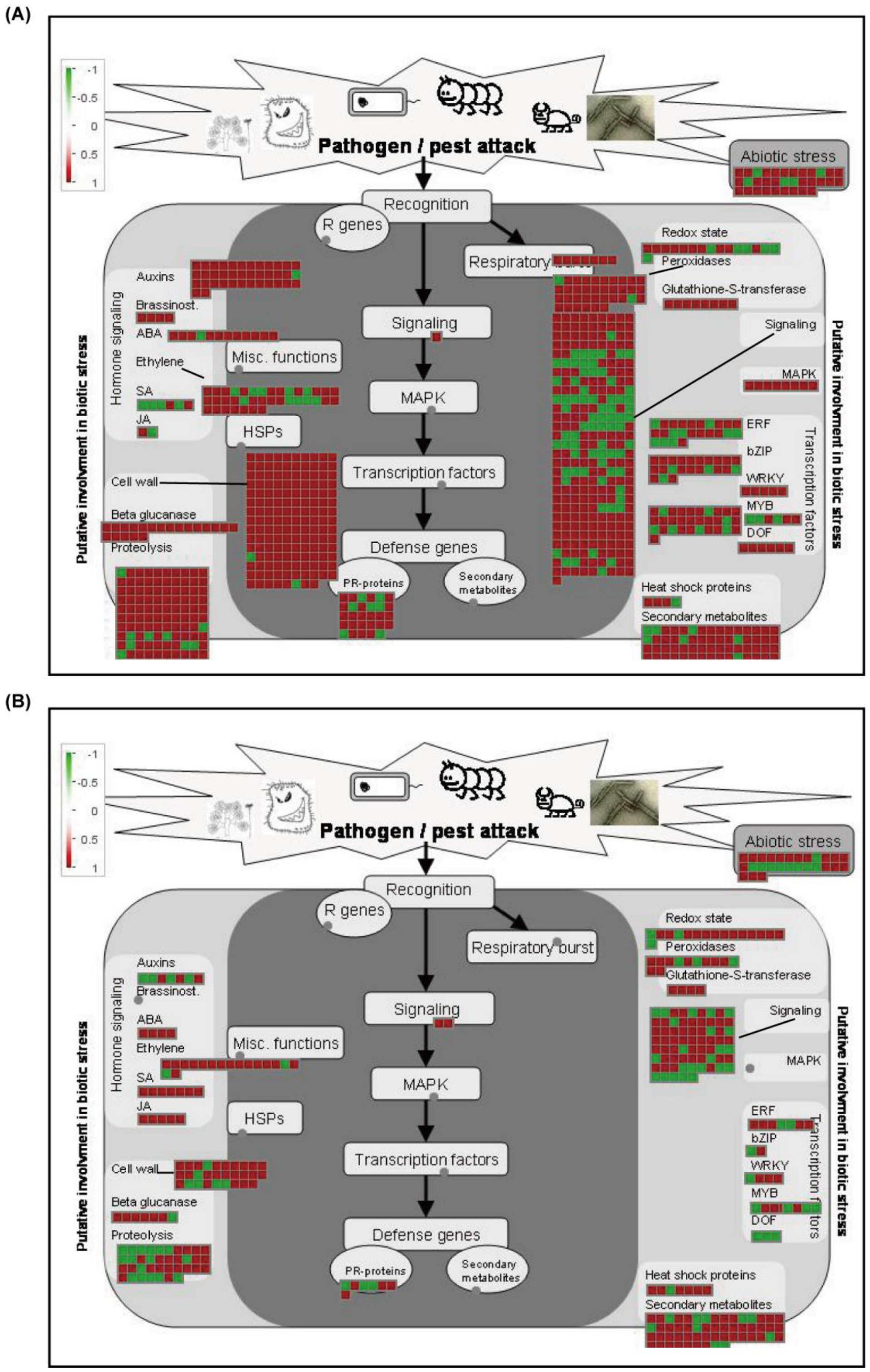

Figure S2. Differentially expressed genes (DEGs) in Nipponbare and C331 plants mapped onto a biotic stress pathway. (A) DEGs in Nipponbare plants that had been fed by BPH for 48h compared with untreated Nipponbare. (B) DEGs in C331 plants that had been fed by BPH for 48h compared with untreated C331. Red boxes represent upregulation, and the green boxes represents downregulation.

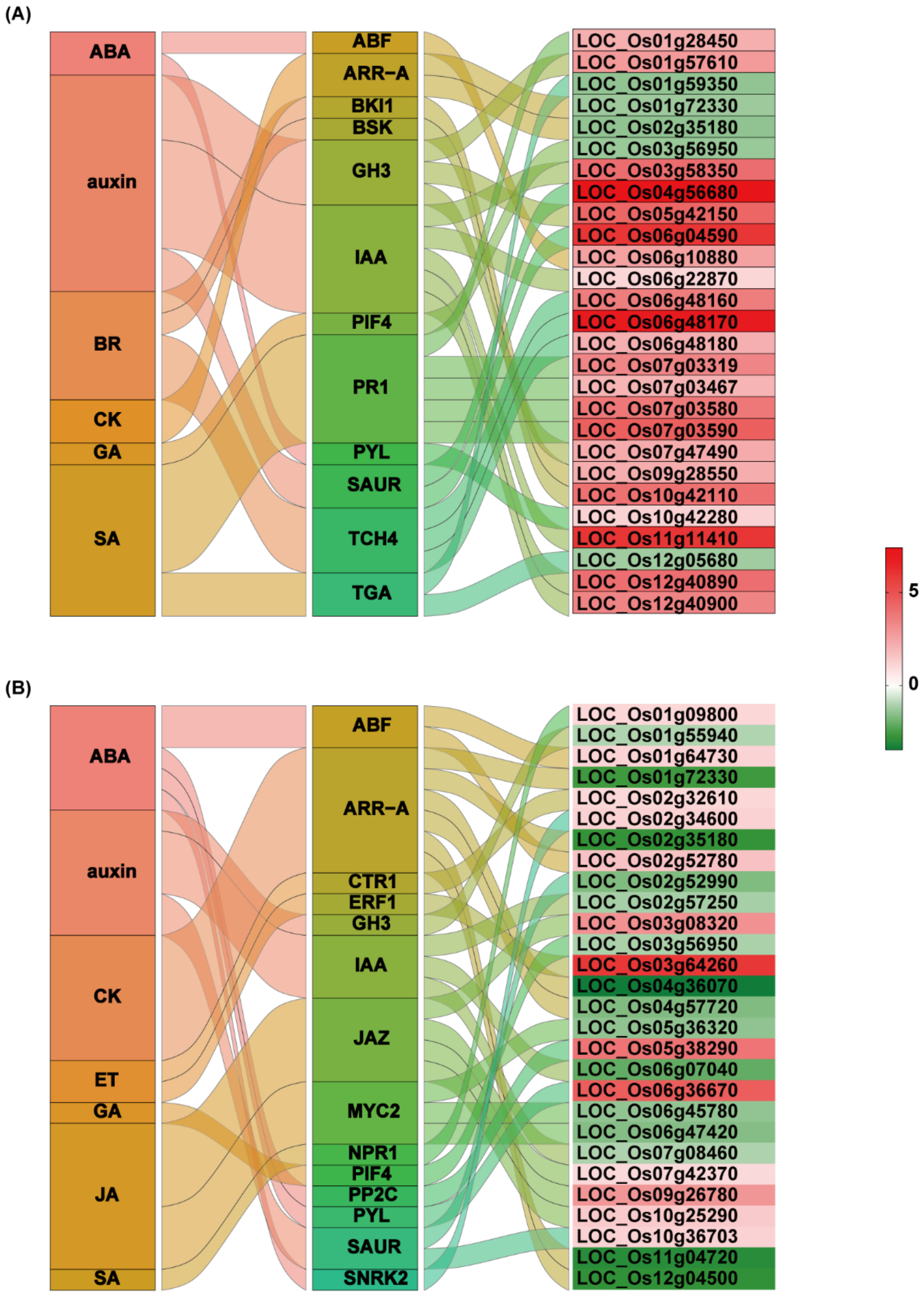

Figure S3. Alluvial map of plant hormone related differentially expressed genes (DEGs) of Nipponbare and C331 attacked by BPH for 48 h. (A) Alluvial map of plant hormone related DEGs in Nipponbare attacked by BPH for 48 h. (B) Alluvial map of plant hormone related DEGs in C331 attacked by BPH for 48 h. Heatmap represents the log2-transformed fold changes of DEGs, red circles represent upregulated genes, and green circles represent downregulated genes. The darker the color, the larger the difference.

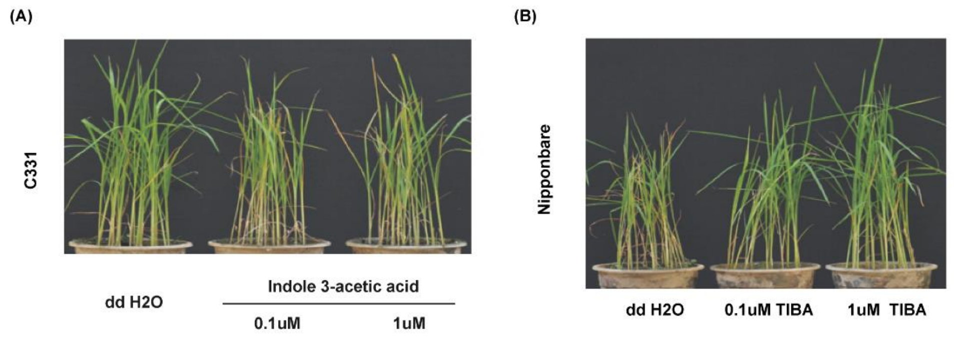

Figure S4. Indole 3-acetic acid (IAA) and auxin transport inhibitor 2,3,5triiodobenzoic acid (TIBA) regulate BPH resistance in rice. (A) A representative image of the C331 plants after pre-treatment with ddH2O, 0.1μM IAA, or 1 μM IAA applied to plant leaf sheaths for 2 hours followed by 10 third-instar BPH nymphs per plant for 4 days. (B) A representative image of the Nipponbare plants after pre-treatment with ddH2O, 0.1μM TIBA, or 1 μM TIBA applied to plant leaf sheaths for 2 hours followed by 10 third-instar BPH nymphs per plant for 3 days.

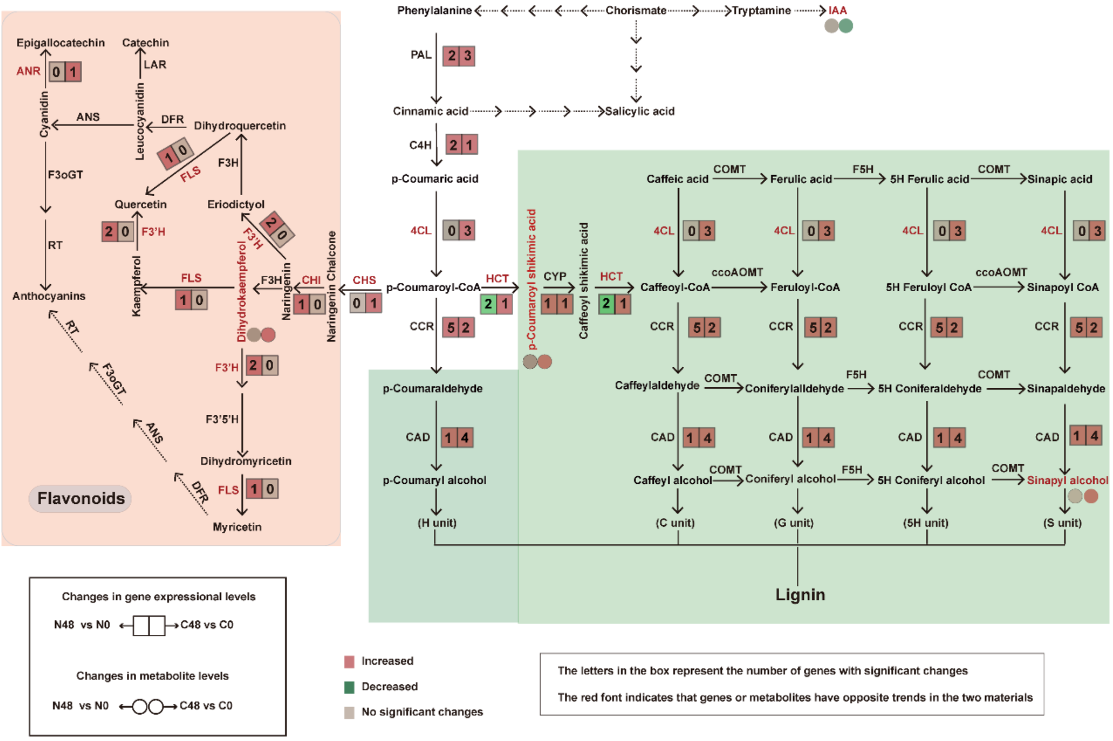

Figure S5. Differently accumulated metabolites (DAMs) and differentially expressed genes (DEGs) in phenylpropanoid metabolism in Nipponbare and C331 fed by BPH for 48h. N0 and N48 indicated Nipponbare fed by BPH for 0h and 48h, respectively. C0 and C48 indicated C331 fed by BPH for 0h and 48h, respectively. The rectangles represent the genes and the circles represent the metabolites. The red color represents upregulation, and the green color represents downregulation. The numbers in the rectangles represent the number of genes significantly changed after BPH infestation.

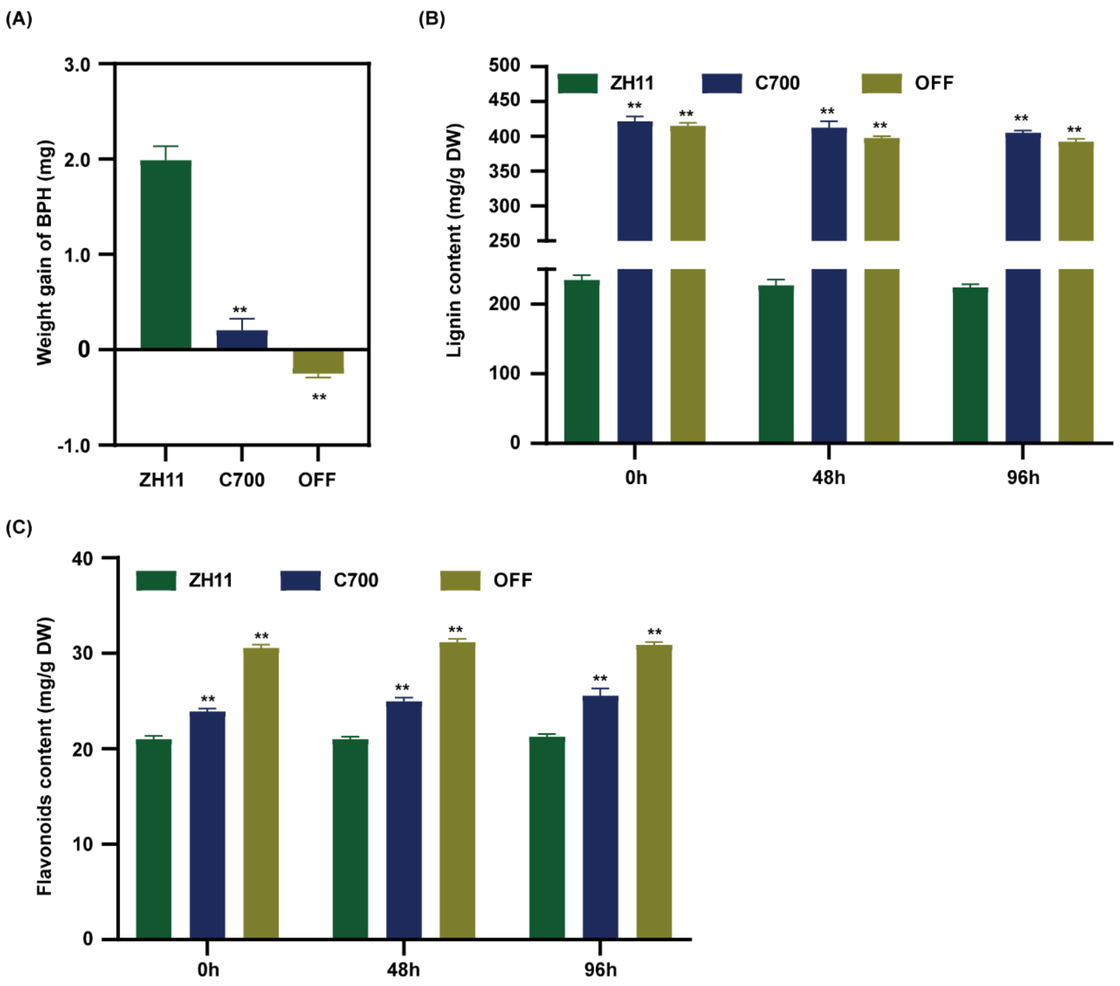

Figure S6. Resistance level, lignin and flavonoids content of another three rice varieties. (A) Weight gain of newly emerged female adult BPHs that had fed on ZH11, C700 and OFF for 48h. Three independent experiments were repeated with 20 BPH insects per replicate. (B) Lignin contents of ZH11, C700 and OFF plants fed by BPH for 0h, 48h and 96h. (C) Flavonoids contents of ZH11, C700 and OFF plants fed by BPH for 0h, 48h and 96h. Error bars, mean ± SE of 3 biological replicates, by students T test, at* $P< 0.05$ (*) and; ** $P< 0.01$ (**), respectively.

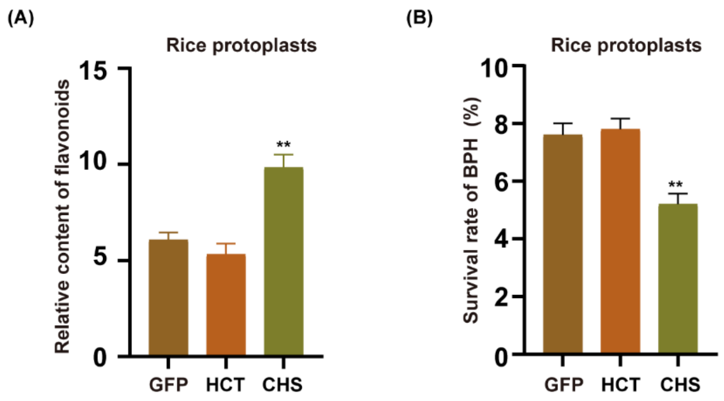

Figure S7. Flavonoids content in CHS and HCT overexpressed rice protoplasts and survival rate of BPH fed on artificial diet supplemented with flavonoids extracted from CHS and HCT overexpressed rice protoplasts. (A) Quantitative determination of the flavonoids contents extracted from CHS and HCT overexpressed rice protoplasts. (B) Survival rates of BPH fed on artificial diet supplemented with flavonoids extracted from rice protoplasts for 4 days. Error bars, mean ± SE of 5 biological replicates, by students T test, at* $P< 0.05$ (*) and; ** $P< 0.01$ (**), respectively.
